# Women’s experiences with continuity for effective coordination during maternal and neonatal continuum in Kenya: An interpretive phenomenology

**DOI:** 10.4102/phcfm.v16i1.4444

**Published:** 2024-08-27

**Authors:** Grace M. Wainaina, Doreen K. Kaura

**Affiliations:** 1 Department of Nursing and Midwifery, Faculty of Medicine and Health Sciences, Stellenbosch University, Stellenbosch, South Africa

**Keywords:** women’s experiences, continuity, coordination of care, maternal neonatal health care, needs, preferences

## Abstract

**Background:**

Embracing women’s experiences in decision-making is imperative for continuity in effective coordination of maternal and neonatal health (MNH); women are the end users within the care ecosystem. Through women’s continuous feedback, skilled birth attendants (SBAs) and the healthcare system get to understand emerging issues based on their needs and preferences.

**Aim:**

The purpose of this article is to describe women’s experiences of continuity for effective coordination of care through the transitions in the MNH continuum in Kenya.

**Setting:**

The study was conducted in selected counties of Kenya based on birth rates per woman as follows: Wajir (7.8) Narok (6.0) Kirinyaga (2.3) and Nairobi (2.7) (1). The clients were interviewed concerning their experiences of the MNH continuum of care in English and Kiswahili.

**Methods:**

An interpretive hermeneutic phenomenological approach was used to construct the experiences of women of continuity during transitions in the MNH continuum for effective care coordination. Twelve participants were interviewed between January and April 2023. Atlas ti 22 software was used for data analysis.

**Results:**

Four women experiences were highlighted: Women unawareness of preconception care, use of prenatal care, labour, birthing and postpartum flow and the women’s view on the MNH continuum.

**Conclusion:**

The women reported their segmental and transitional experience of the MNH continuum as one that did not consistently meet their needs and preferences in order for them to fully agree that the continuum enhanced continuity for effective coordination. They felt that they experienced continuity in some segments while in some they did not.

**Contribution:**

The embrace of women’s experience of their needs and preferences through the MNH continuum (segments and transitional segments) through the lens of continuity for effective coordination is timely towards the improvement of maternal and neonatal care by 2030.

## Introduction

Women’s experience of continuity and coordination of the maternal and neonatal health (MNH) continuum has at times been highlighted as dissatisfaction. Some of the dissatisfaction includes women’s lack of confidence during the postpartum period,^1^ a lack of respectful care during birthing, labour and delivery.^2^ It is therefore important that the woman is given a chance to frequently give feedback on her experience of continuity for effective coordination, as she transitions through the segments. This is because she is the consumer of the service. The researcher hopes to encourage the Skilled Birth Attendant’s (SBAs) to appreciate, uphold and embrace women’s voiced experiences where they (women) are allowed to express their needs and preferences of the continuum. The SBA should appreciate that these same clients need their (midwives’) knowledge and advice and would never want to be viewed as ‘demanding clients’ but rather as good-willed and contributing stakeholders in the continuum.

This article is part of a series aimed at facilitating the development of evidence-based guidelines for coordination and continuity of care through the transitions (preconception, pregnancy, birth and postpartum period) in the MNH continuum in Kenya. The first phase of this development involved conducting a qualitative synthesis of SBAs’ experiences of continuity for effective coordination of the MNH continuum. The second phase involved listening to the SBAs verbalise their experiences as they cared for women throughout the MNH continuum. Notably, studies on continuity of care from SBAs are abundant in the literature.^3,4,5^ However, according to the researchers’ knowledge, little is known concerning the women’s voiced experiences of their needs and preferences as they transition through the MNH continuum. This article (phase three) therefore aims to reveal the experiences of women as they transition along the MNH continuum through the lens of continuity for effective coordination. The combination of women’s feedback as well as SBAs’ feedback is of great importance to the development of evidence-based guidelines.^6^ Therefore, as their experiences were sought, WHO^7^ and Radwin’s^8^ frameworks were utilised to discuss the findings.

## Research method and design

### Study design

An interpretive hermeneutic phenomenological approach^9^ was used.

### Study setting

The study was conducted in Kenya, in four facilities from four selected counties namely: Pumwani Maternity Hospital (A City Public Government Hospital-Level 5) from Nairobi County (second lowest fertility rate 2.7 births per woman); Sagana Catholic Dispensary (A Faith Based Health Facility-Level 2) from Kirinyaga County (lowest fertility rate of 2.3 births per woman); Ewasongiro Health Center (A Rural Health Centre-Level 3) from Narok County (the second highest fertility rate of 6.0 births per woman) and Bute Subcounty Hospital (A Rural facility Sub-County-Level 4) in Wajir County (highest fertility rate of 7.8 births per woman).^1^ During the selection of counties and facilities, various factors were considered namely: the level of facilities in relation to vertical and horizontal coordination of facilities from one level to another, where Level 1 comprised the community facilities that handled minor ailments and basic MNH services to mothers in the community. Level 2 facilities were health dispensaries that received referrals from Level 1 and could refer to subsequent higher levelled facilities for continuity and coordination of care. The fertility rate of the counties was also sought so as to enhance the availability of the participants at various levels while also considering geographical settings, government and or public sponsored facilities as well as private faith-based and non-faith-based institutions.^6^

Women who were in the postpartum period (24 h to 2 weeks post-partum) were eligible for the interview.

### Study population

The study population included mothers of reproductive age between 19 and 49 years. The mothers were selected from four healthcare facilities that catered for all the six healthcare facilities’ considerations. Mothers were recruited in their respective facilities on their experience of continuity for effective coordination as they transitioned along and in between the segments of the MNH continuum. A non-probability purposive sampling strategy was adopted. The recruitment and interviewing process commenced from January 2023 to May 2023. Twelve women participated in the study. In Pumwani Hospital, four women participated, in Sagana Catholic Dispensary, one woman, in Ewasonyiro Health Center, four women and in Bute Subcounty Hospital, three women. Saturation was achieved at the 12 participants.

### Sampling

Non-probability purposive selection was conducted among 12 women who had just delivered (24 h to 2 weeks post-partum, representing first target postpartum contact to second target postpartum contact after delivery) so that they could describe their experience of the MNH continuum.

### Data collection

The in-depth interviews with the 12 participants were conducted between January and May 2023 and audio recordings were obtained. The in-depth interview guide had been developed after conducting the systematic review (phase 1). The guide was pretested with three women from a different study setting. The interview guide statements were then modified, simplified and then utilised. The in-depth interviews were conducted in a room that was allocated by the hospital in charge and they lasted between 30 and 50 min. Once the recordings were completed, the Kiswahili recordings were transcribed into Kiswahili and the Kiswahili transcripts into English by the appointed language expert. The primary researcher also reviewed the transcriptions. The transcriptions were then cleaned (by the principal investigator) and uploaded into Atlas ti (version 22) for coding. Codes were created inductively and code groups developed. Meanings were later obtained from the code groups.

## Results

### Demographics

All 12 participants were married. They were in their 20s and 30s; some of them were students who dropped out of school because of pregnancy, and some were professionals. The women were mothers of between one and four children. Table 1 highlighting the demographics of women from four facilities in Kenya.

**TABLE 1:** Demographic information for the women.

Women registration	Marital status	Age (years)	Highest education attained	Parity	Occupation (at the point of interview)
Pumwani woman 1 (PW1)	Married	24	College	2	Housewife
Pumwani woman 2 (PW2)	Married	20	High school	1	Housewife
Pumwani woman 3 (PW3)	Married	39	Primary school	2	Hairdresser
Pumwani woman 4 (PW4)	Married	30	High school	1	Housewife
Bute woman 1 (BTW1)	Married	29	College	3	Teacher
Bute woman 2 (BTW2)	Married	24	Primary school	3	Housewife
Bute Woman 3 (BTW3)	Married	20	High school	2	Housewife
Sagana woman (SGW1)	Married	30	College	2	Medical Records officer
Ewasonyiro woman 1 (EW1)	Married	20	Primary school	1	Housewife
Ewasonyiro woman 2 (EW2)	Married	38	Primary school	3	Casual labourer
Ewasonyiro woman 3 (EW3)	Married	20	High school	4	Farmer
Ewasonyiro woman 4 (EW4)	Married	24	High school	3	Housewife

The following are meanings that have been derived, based on emerging experiences (Table 2).

**TABLE 2:** Experiences of women on continuity for effective coordination of care.

Meanings	Emerging experiences
Women unaware of preconception segment	Preconception care (PCC) is stopping a contraceptive once married
	No need for supplements; just wait to conceive
Staying pregnant and being active	Some pregnancies were planned and prepared and others unplanned and unprepared
	How they accessed prenatal care
	Good reception and prenatal services offered
Laboring, birthing and postpartum flow	Timely access to labour and birthing segment
	The encounter and staying strong after birth
	Discharge and postpartum follow-through plan
The women’s view on the MNH continuum	A lack of valuing women’s preferences
	No partnership in the MNH continuum
	The unaddressed needs of women
	What the women liked

MNH, maternal and neonatal health.

### Women unaware of preconception segment

When asked about their experience during preconception care (PCC), some women were not aware that this was an important segment. They were not aware of the exact timing or activities during this segment.

#### Preconception care is stopping a contraceptive once married

The women described PCC as a time, after marriage, when they just withdrew from using a contraceptive in order to conceive:

‘I just stopped (taking contraceptives). I didn’t use any medication … [*f*]irst I was not using any family planning methods or contraceptive …. I didn’t have a reason to prepare because I was still a student …’ (Interviewee PW1, 24 years old, Housewife)

#### No need for supplements, just wait to conceive

The women stated that they did not consume supplements but just ate healthily. They also stated that they did not seek PCC advice because it was shameful. They just waited till they conceived. The waiting period lasted between 2 months and 5 years:

‘I did not use any supplements apart from just eating healthy.’ (Interviewee SGW1, 30 years old, Medical Records Officer)

‘They (women) are very ashamed to say or ask that. You can’t do that. You just go ahead and conceive.’ (Interviewee BTW1, 29 years old, Teacher)

‘Once, I was advised by a fellow mother to go get hormonal medicines but I ignored the advice. Later, after 5 years, it’s when now I conceived.’ (Interviewee PW3, 39 years old, Hairdresser)

The PCC segment seemed to be a private and less publicised segment. To women, it was shameful to seek advice. It seemed obvious that they just needed to stop using contraceptives and continue having unprotected sexual relations until they were pregnant. They did not need to tell SBAs or other people that they were trying to conceive. They just waited for months or years till they conceived.

### Staying pregnant and being active

Some women reported to have prepared and planned for their pregnancy while others did not. Once the women realised that they were pregnant, they accepted their status. Notably, the majority accessed prenatal or antenatal care in the second trimester. Among those who accessed this care, they reported a good reception throughout the segment.

#### Some pregnancies were planned and prepared and others unplanned and unprepared

The women who reported that they had planned and prepared for their pregnancy were happy when they conceived, while those who considered their pregnancy to be unplanned and unprepared described it as an accident and acknowledged being stressed. However, both groups later accepted their pregnancy and moved on:

‘… [*l*]ater, after 9 years, it’s when now I conceived and I was happy.’ (Interviewee PW3, 39 years old, Hairdresser)

‘I hope to have a plan next pregnancy and not just by accident …’ ‘When I discovered that I was pregnant, I was stressed.’ (Interviewee PW2, 20 years old, Housewife)

‘Now that I have conceived, … I didn’t see the need to abort. I just decided to keep the pregnancy.’ (Interviewee EW4, 24 years old, Housewife)

#### How they accessed prenatal care

Pregnant mothers in the urban areas accessed a prenatal clinic that was close to them, whereas mothers from marginalised areas moved close to the health facilities, leaving behind their families for their relatives to take care of. Notably, most women delayed prenatal access until the second trimester, because either they did not know that they were pregnant, or their relatives or friends advised them to do that, or they just did not want to come early. Late prenatal access led to reduced visitations:

‘I missed [*my*] period on the 4th month, … I self tested and confirmed that I was pregnant.’ (Interviewee PW3, 39 years old, Hairdresser)

‘(I started clinic) When I was 4 months pregnant … The reason is because during these prior months, I normally vomit, I am usually unable to eat … I was told to stay close to the hospital and not to go far.’ (Interviewee BTW1, 29 years old, Teacher)

‘When you ask your relatives friends they all tell you to start (antenatal clinic) at 6 months, so I believed them.’ (Interviewee PW4, 30 years old, Housewife)

‘I don’t have a reason (for delay) … [*I*] just thought that I should start (antenatal clinic) when I am 5 months.’ (Interviewee PW1, 24 years old, Housewife)

It seemed women were not concerned about early prenatal access. Probably, they lacked knowledge of its advantages.

#### Good reception and prenatal services offered

When the women accessed the clinics, the following services were offered: issuance of the antenatal booklet, investigations, acquiring their demographic information, health teaching on danger signs, birth preparedness, saving of money and nutritional advice. Unfortunately, some mothers stated that they were never taught about danger signs:

‘On accessing the clinic, I was well received by the nurse … She gave me the antenatal booklet for the clinic, and she sent me to do some tests … I was told if I see bleeding, or if my legs swell, when I get dizzy, if I feel my heart beating a lot, I should come. So she told me to be saving some 100 to 200 shillings … and have a home bank for emergency.’ (Interviewee BTW1, 29 years old, Teacher)

‘I was told that I should carry some diapers, baby clothes and also carry one or two clothes for myself.’ (Interviewee PW3, 39 years old, Hairdresser)

‘They only told us to read a certain page on danger signs.’ (Interviewee PW1, 24 years old, Housewife)

During the antenatal period, women were keen to acknowledge their husbands’ support. They requested them to accompany them to the clinics so that they could understand the care they received:

‘So then next time I was to go to the hospital, I asked him to come with me so that he could understand what was going on … as the father of the baby decided that he will cater for his child and myself.’ (Interviewee PW2, 20 years old, Housewife)

It was encouraging that all women accessed prenatal care and they were empowered with information. However, the late prenatal access was significant because it was as a result of knowledge deficit about the importance of early access, and it led to reduced interaction between the SBAs and the women.

### Labouring, birthing and postpartum flow

Women reported both scheduled and unscheduled labour. They stated that they accessed the labour segment after experiencing alert or danger signs. After the labour segment, some accessed the birthing segment on time while a few accessed it late. Each of them described their first encounters with their newborns. They also stated how they stayed strong after birth and accessed the postpartum transition and segment.

#### Timely access to labour and birthing segment

Once the women experienced alert or danger signs, they accessed the labour wards. Some of the alert signs they experienced were: bleeding, pain, headaches and water breaking:

‘I was at home when the pain started. The pain was coming and disappearing, so I had to come to the hospital because it was not normal.’ (Interviewee PW1, 24 years old, Housewife)

Notably, the women acknowledged that they responded faster during pain alerts than any other alerts:

‘[*M*]y water broke … I did not have an idea of what to expect or do; then later on I started having pain; then I went to the hospital … the labor started earlier than the date I had been given.’ (Interviewee SGW1, 30 years old, Medical Records Officer)

Upon arrival in the labour wards, the women were well received, assessed, reviewed and given feedback on the progress of labour:

‘I came using a vehicle up to here … I came and found the doctor and he attended to me … When he tested me, he told me I was almost; I stayed for a while; I didn’t stay for long.’ (Interviewee EW2, 38 years old, Casual laborer)

‘When we came to the hospital, we found the doctor; we were received well, I was reviewed.’ (Interviewee BTW1, 29 years old, Teacher)

Throughout the labour period, the women appreciated efforts to enable them to cope with pain:

‘I had someone who at least was near me to help rub my back.’ (Interviewee SGW1, 30 years old, Medical Records Officer)

‘What made me happy was … the injection helped reducing pain during birth … The one injected at the back … That is okay, they should not remove.’ (Interviewee PW1, 24 years old, Housewife)

#### The encounter and staying strong after birth

After birthing, mothers appreciated being shown their newborn immediately; some mothers reported discontent when they were not shown their newborn immediately. The delay in the first encounter was because of the prioritisation of newborn resuscitation or urgent help:

‘I was shown my child immediately.’ (Interviewee BTW1, 29 years old, Teacher)

‘So the baby was born and I was shown and I identified it was a boy …. It was beautiful; the baby was chubby; it was nice.’ (Interviewee SGW1, 30 years old, Medical Records Officer)

‘Then later, I was told that I gave birth to a baby girl who had been taken to the nursery because the baby had a distended abdomen.’ (Interviewee PW4, 30 years old, Housewife)

After birthing, the women realised that some procedures had to be carried out even if they were uncomfortable. They also acknowledged that they were given food and drink after birthing. This was the first step of postpartum:

‘They removed the placenta … then cleaned [*me*] … to remove all the clots so that I don’t get problems in future … After birthing, I asked for painkillers because I was having a headache and she (SBA) gave me paracetamol. … Then I was given porridge.’ (Interviewee BTW1, 29 years old, Teacher)

‘You know, for me blood was coming out a lot. So the cleaning was painful and necessary.’ (Interviewee EW4, 24 years old, Housewife)

#### Discharge and postpartum follow-through plan

Mothers who delivered through spontaneous vaginal delivery were discharged after 24 h, while those who delivered through caesarean section were discharged after 3 days. Mothers and children who had complications were retained for at least 3 days. Once the mother and the baby were stable, they were discharged and given a 2-week return date for the second postpartum visit. Some of them knew the reasons for their 2-week return, while others did not. In fact, some wondered if postpartum visits were as important as antenatal visits:

‘I stayed for almost 3 days because I was being monitored … (after discharge). I didn’t t know what we were coming to do or what we could be checked for … we were just coming … We then came back after 2 weeks, and when we came they checked his weight and he was 5.6 kgs.’ (Interviewee SGW1, 30 years old, Medical Records Officer)

‘Is it a must I take the baby to clinic just as I was doing during the pregnancy period?’ (Interviewee PW2, 20 years old, Housewife)

As the mothers prepared to be discharged, they were asked about plans for the next conception. The responses ranged from the immediate commencement of a contraceptive to an unidentified later date:

‘I would like to start with injection (contraceptive) today.’ (Interviewee PW3, 39 years old, Hairdresser)

‘I am not intimate with my husband and he is not at home. He understands that I am still healing. So I will start later.’ (Interviewee PW1, 24 years old, Housewife)

The women’s lack of awareness of the postpartum content and commitment revealed their ignorance of the postpartum segment. It seemed that once the baby was born, mothers felt that it was the end of caution. Before mothers were discharged to go home, they were asked about their next preconception plans. Some were receptive to commencing a contraception plan while others thought it was too early.

### The women’s view on the maternal and neonatal health continuum

The women were given an opportunity to describe what they liked or disliked during and along the MNH continuum. Notably, they described what they disliked in their preferences while their likes seemed to have been captured in their positive experiences during the continuum. As they described their experiences, they seemed to prefer proper healthcare financing, enhancement of health workforce, availability of medical products and a well-structured leadership, governance and service delivery.

#### A lack of valuing women’s preferences

As the women transitioned through the continuum, they highlighted the need to enhance the nature of the workforce that they preferred: they preferred women SBAs over male SBAs:

‘I would like that (women nurses) … although I don’t mind the male nurses too.’ (Interviewee BTW1, 29 years old, Teacher)

#### No partnership in the maternal and neonatal health continuum

Some mothers felt that there was no partnership between SBAs and themselves. It seemed that what the SBAs said was not to be questioned but just followed. They stated that they were made to sign consents when in pain. They felt manipulated:

‘Then the pains increased … the whole body was in pain. I was later told that I would go for a CS [*Caesarean section*] … that’s when I was given some papers to sign and write my name … I filled in the date too and was told to read the rules. I just read in general.’ (Interviewee PW4, 30 years old, Housewife)

One woman did not want to sign for a caesarean section because she was not consulted. She stated that she wanted to try delivering through spontaneous vaginal delivery:

‘I came and the one who received me told me … since I do CS [*Caesarean section*] I should be booked first. I struggled and debated with the nurses a bit since I didn’t want to be checked and booked for CS [*Caesarean section*]. So I stayed on that day 10th and the pain got worse finally decided to go for CS [*Caesarean section*] on 11th.’ (Interviewee PW3, 39 years old, Hairdresser)

#### The unaddressed needs of women

The mothers reported displeasure when their needs were not met. For example, unavailability of drugs, paying cash for health services despite being enrolled in a health scheme and rude SBAs who were not approachable for discussion about their newborn’s progress:

‘So I had to pay … even if I had enrolled for Linda Mama.’ (Interviewee PW1, 24 years old, Housewife)

‘Everything was okay except from New Born Unit [*NBU*], I didn’t know what was the baby’s problem. I was always worried. … [*t*]he doctors never told me the exact problem of the child … There is one doctor who talked to me so rudely … I was concerned because the baby was crying for long … [*t*]here wasn’t enough time to see the baby in the nursery room, so you would come and see the baby but no one told you how the baby was doing or progressing. So because the time in NBU is short, you go back if not satisfied.’ (Interviewee PW4, 30 years old, Housewife)

‘I wish they had enough drugs so that we don’t go looking for drugs all over.’ (Interviewee PW2, 20 years old, Housewife)

#### What the women liked

Mothers appreciated when SBAs taught them, accompanied them during referrals in hospital transport and allocated community stakeholders to follow them up. They also liked it when they were given a chance to describe the continuum as they viewed it, to ask questions and to express their needs, experiences and preferences concerning healthcare:

‘I didn’t know how to breastfeed until a nurse came and showed me how to.’ (Interviewee PW4, 30 years old, Housewife)

‘[W]e were taken by an ambulance … 2 nurses from here. One of them helped me carry the baby and the other one was carrying the oxygen. We were received immediately … It’s like they had communicated. The baby was taken inside for the scan, though I was scared about the whole process.’ (Interviewee PW4, 30 years old, Housewife)

‘I have noted that there are some people from the health facility who call themselves community volunteers. Those are the ones whom the government sends to us to give health messages (Nutrition, conception, immunization).’ (Interviewee BTW1, 29 years old, Teacher)

The reaction of the mothers when they were given a chance to ask a question unveiled an unmet need that they had for an interactive relationship with their SBAs. This relationship needed to be cultivated and encouraged for continuity and coordination of care.

## Discussion

The experiences of the women as they transitioned through the MNH continuum of care revealed either some form of continuity for effective coordination or at times a lack of it. It is important to listen to the women as they are the consumers of the continuum. They highlighted meanings based on their experiences. They included: access to the unknown PCC segment, staying pregnant and being active, the labouring, birthing and postpartum flow, and their view on the MNH continuum.

### Women unaware of preconception care segment

The women’s view on PCC seemed simplistic and it exposed their lack of awareness. This was because, out of all the 13 components of PCC, they only cited: withdrawal from a contraceptive in order to conceive, avoidance of consultation during preconception time, no need for supplements and commencement of the process of conceiving after marriage. This limited level of PCC awareness would have been because of the lack of exposure to correct PCC information, as well as a lack of knowledge of the segments’ relevance for both the SBAs and the women.

Notably, PCC contains 13 components that the woman needs to experience, namely nutritional assessment, cessation of tobacco use, screening of genetic conditions, environmental health, screening and management of infertility, interpersonal violence, preventing unwanted and rapid successive pregnancies, treatment of sexually transmitted infections (STIs), prevention and management of HIV, mental health, prevention of psychoactive substance use, immunisation and prevention of female genital mutilation.^11,12^

The women had limited experience with PCC. This was observed when they stated PCC as the discontinuing of a contraceptive in order to conceive. When their experiences are viewed in the light of what ought to be the ideal PCC experience, their experience is limited and incompatible with the results of other studies.^13^ A comparison between what the women experienced with what PCC ought to be, revealed gaps that can lead to fragmentation of care in low- and middle-income countries, as previously cited.^14,15^ It is therefore important that the women are encouraged by SBAs to embrace all recommended PCC components.

In addition, the women stated that they never prioritised supplementation during PCC, but rather assumed that diet was sufficient. However, emphasis on preconception supplementation was advised for improved birth outcomes^16,17^ and child intellectual ability till the age of six.^18^ Therefore, it was important to appreciate that sometimes diet would not be balanced and sufficient, so supplements were important.

Also, the women stated that PCC should start after they are married. This was not advisable, as some adolescent mothers conceived early and later described their pregnancies as accidents. Therefore, the ideal way is to make PCC accessible to all women of reproductive age, to evaluate risks and promote health interventions and psychosocial health.^10,13,19^ This lack of early reproductive health information was probably because of SBAs’ lack of awareness that it was their role to communicate PCC information to women.

Some women revealed that, as they tried to conceive, they avoided consultations because of shame. Notably, mothers sought PCC guidance without fear or shame if they thought SBAs were friendly and experienced.^20^ At times, women decided to be silent as a defence mechanism against disappointment and false expectations of themselves and their spouses.^13^ It is therefore important for the women to feel reassured concerning their fertility, that whether they conceive or not, or wait for long or short periods, access to continuity for effective coordination on the MNH continuum was availed.^7,8^ It is the role of the SBAs to avail this information for women’s consumption.

The women also stated that they adopted a ‘wait and see’, passive delay period during PCC, for months to years. This period sometimes led them to re-seek advice from SBAs, their friends and relatives or just stay on their own. This was the transitional period towards conception. Notably, no literature to the best of researchers’ knowledge was found to reference this part of the segment. However, this study would like to acknowledge the waiting season as a transitional season that should be addressed by SBAs by communicating all the PCC content until a mother conceives. Even if women are passive during PCC, they still need SBAs.

### Staying pregnant and being active

Staying pregnant and being active meant that women accepted their pregnancies, whether planned or not planned. This segment revealed how the women accessed the prenatal care and the reception they received as they transitioned through the segment.

Women who planned and prepared for their pregnancies reported being happy and content, while those who did not plan and prepare described their pregnancies as accidents and stressors. Previously, in other studies, women with unintended pregnancies reported mental instability delayed prenatal onset, poor nutrition as well as suicide attempts.^21^ This justifies the need for early reproductive health education at the beginning of the reproductive years to encourage women to prepare and plan for their pregnancies.

After conception, early prenatal access was dependant on when the women knew they had conceived. Some did not know that they were pregnant until they were 12–16 weeks, which was highlighted as late pregnancy recognition. Women who had late pregnancy recognition risked compliance with healthy habits and complications for the child and the woman.^22^ This late pregnancy recognition should therefore be avoided by issuing early reproductive health education for the enhancement of a well-planned pregnancy.

As a result of late pregnancy recognition, late prenatal access was noted 4–9 months into pregnancy, despite good SBA reception. This was a different finding from other studies in which late prenatal access was associated with rude SBAs who did not observe confidentiality.^23^ However, similarities of late prenatal access were noted with unintended unplanned pregnancies, hyperemesis gravidarum, relatives and family advice.^24,25^ Notably, hyperemesis gravidarum was cited by others as a reason for early access,^26^ but the women in our study cited it as a reason for delayed access because they felt too weak to access healthcare. Late prenatal access reduced the number of prenatal care visits.^27^ The late entry to segments that led to reduced interactions between SBAs and women is suggested in this research to be a hindrance to both informational and relational continuity.

As the women transitioned through the prenatal segment, each one accessed at least one visit. This confirmed Kenya’s overall figure of 92.8% prenatal access^28^ and was much better than the global figure of 64% access.^27^ This compliance with the Kenyan antenatal guideline probably revealed that the women found prenatal care to be: simple, safe, friendly, accessible, personalised, enhancing their rights, dignity, privacy and confidentiality, and giving access to full and accurate information.^29,30^ This access revealed that the women acknowledged skilled birthing services. This knowledge can be maximised when they are made aware that their feedback on continuity and coordination of care also matters, especially when they express their needs and preferences.

It was also observed that as the women transitioned through the prenatal segment, those in the urban areas accessed antenatal clinics that were near their homes, while those in marginalised areas made an extra effort and moved several kilometres to access clinics and birthing facilities, leaving behind their families. The location of a woman’s residence did not seem to negatively affect her prenatal access or utilisation. This was in contrast to previous highlights, where women’s close residential proximity to the facility facilitated their access to healthcare and improved their health and quality of life during the prenatal segment.^31,32^ Notably, the fact that women travelled short and long distances to access a health facility meant that they valued healthcare and their pregnancy.

Once the women accessed the prenatal segment, they acknowledged having received the following services: investigations, screening, immunisations, physical assessment, treatment of ailments, health teaching on danger signs, birth preparedness and finances. Notably, among the information given, women noted laxity in health education about danger signs. This agreed with findings that some mothers received danger signs education during prenatal segments while others did not.^33^ This is unacceptable because mothers who are unaware of danger signs lack comprehensive birth preparedness and knowledge of transiting to the next segment.^34^ This calls for better and more frequent communication of danger signs information from SBAs to women.

### Labouring, birthing and postpartum flow

The transition and access to the intrapartum segment was a result of the response to alert or danger signs. Notably, women responded swiftly to pain, whereas those who had rupture of membranes delayed accessing care until the pain started, therefore risking contraction of infections.^35^ It was observed that women ranked alert signs. This ranking seemed to determine how fast they sought care from a SBA. This understanding needed to be corrected because all alert or danger signs are important and warrant an urgent response.

Alert signs signalled the commencement of labour. Notably, women were concerned that labour commenced either on the expected date of delivery (EDD) or earlier than expected. Their concern was that what the SBAs told them about EDD was at times not ‘correct’. It was therefore vital to educate them that early labour onset could be the result of a number of factors, namely placental production of the peptide corticotrophin releasing hormone (CRH) that altered the length of gestation, a previous history of prematurity,^36^ low antenatal care utilisation, premature rupture of membranes because of infections, malaria, hypertension in pregnancy, maternal age of less than 20 years or more than 35 years.^37^ It is therefore important for the women to be well informed during antenatal visits about birth preparedness.

As the women progressed through intrapartum, pain was their real concern. However, they appreciated efforts from their relatives or birth partners, who helped relieve the pain by massaging their backs and supporting them, as previously reported.^38^

As the pain increased and the mothers transitioned to birthing, they appreciated immediate encounters with their newborns. Unfortunately, some mothers reported delayed first encounters, yet this moment was a pinnacle starter point of the child-bearing process that stabilises the child’s vital signs, reduces crying and enhances bonding.^39^ After birthing, the women accessed and stayed in the postpartum segment from 24 h to 3 days, before home discharge. This was in line with postnatal guidelines concerning the interval stay.^40^ Apparently, all mothers accessed the first postnatal visit (24–48 h) and a few of them accessed the second (2 weeks after birth). Some mothers wondered if the postpartum visits were important enough to follow through with the third visit (4–6 weeks) and fourth visit (4–6 months), as advised in the target postpartum care (TPPC) Guidelines.^40^

As the women transitioned and utilised TPPC, some of the components to be accessed were: assessment of the baby, cord care, counselling and support offered to the breastfeeding mother, postnatal care for the baby, assessment of the mother, counselling of danger signs and family planning advice, iron and folic acid supplementation, prophylactic antibiotics for those women who required them and psychological support.^40^ Notably, the majority of the women delivered in facilities with SBAs, but experienced their first TPPC elsewhere as previously highlighted.^41^ It was important to observe that the women had limited experience with TPPC as well as its importance. This therefore was seen as a potential hindrance to continuity for an effective MNH continuum.

### The women’s view on the maternal and neonatal health continuum

As the women transitioned through the continuum, they felt that their preferences were not valued, and that affected their partnership with the SBAs. They were also concerned that their needs were not addressed, although they mentioned some good experiences.

Firstly, the women stated that they preferred being attended by female SBAs throughout the MNH continuum over male SBAs, because of their cultural or religious orientation, as stated elsewhere.^42^ Therefore, SBAs should listen and pay attention to women’s stated preferences so as to promote respectful communication.^43^ The implementation of women’s preferences can therefore enhance continuity for effective coordination.

Secondly, the study’s findings highlighted that women who had previously delivered through caesarean section did not appreciate when they were described as ‘once a caesarian section mother, always a caesarian section mother’. They wanted to be told the indications of subsequent caesarian section, to be consulted when making decisions, and to be given a chance to attempt a vaginal birth after a caesarean section (VBAC). Notably, VBAC would be considered if the SBAs were skilful, the mother had support and a continuity model like the midwifery care model was implemented.^44^ Relational continuity was therefore important for the facilitation of this intervention.

Thirdly, women reported their disappointment when some of their needs remained unaddressed. They stated that they paid for services at the point of delivery, yet they had been enrolled in a health insurance financing scheme (Linda Mama programme). They claimed that they were informed that, after scheme enrolment, they would not pay cash. The women’s claims were confirmed as the scheme had been suspended at a national level but information had not been communicated to the women in the facilities. The scheme’s suspension was because of challenges in reimbursement logistics to facilities and therefore reverted to out-of-pocket payments.^45^ Therefore, the lack of communication seemed unfair to the women who would have negatively affected continuity of care.

Fourthly, mothers highlighted their disappointment over the unavailability of drugs in the facilities and sometimes a lack of information concerning their health. This was against the Kenya National Patients’ Rights Charter (2013) that outlines the right to access healthcare, concerning drugs, respect and dignity, the right to information and the right to complain.^46^ It is therefore important that the SBAs are made aware of these laws for the enhancement of relational continuity.

However, as the women transitioned through the MNH continuum, some were keen to acknowledge good services and point out the positive experiences they encountered. The women pointed out that there were times when SBAs taught them about breastfeeding, allocated them community volunteers to reinforce PCC, accompanied them during referral, and gave them a chance to ask questions and for the first time to describe what they thought needed to change in the continuum. They also appreciated their men for supporting them financially, emotionally or physically, as they transitioned through the continuum. Male involvement was acknowledged as one that enhanced care-seeking behaviour,^47^ and improved home care practices, communication between spouses and decision-making throughout the continuum as well as birth spacing.^48^ Integration and advocacy for policies and strategies that can improve men’s knowledge in maternal care should therefore be enhanced.^49^

### Strengths and limitations of the study

Firstly, the strengths of this study lie in including women participants from four out of six levels of the Kenyan Health Care System. The selection of these women in different facilities attempted to incorporate all classifications of the Kenyan Health Care System categorisation. This was important towards the formation of a reference point in relation to the objective of the study. Secondly, the early involvement of women (before memory lapse) in the postpartum segment to describe their experience of the MNH continuum seemed ideal so that they would offer a clear and detailed experience of the cycle before discharge. Notably, the woman’s feedback would also assist the SBA to look back and see what in the continuum needed to change before proceeding to the next continuum a few years later. A limitation that might have been experienced was a lack of inclusion to the highest level (level six) of the Kenyan Health Care System Categorisation.

### Implications of the findings

The study’s finding suggest a relook of all the guidelines of the MNH continuum through the lens of continuity and coordination of care so as to reduce fragmentation of care as we proceed to the sustainable development goal (SDG) achievement of 2030. This suggestion is also to embrace the intersegment phases as equally important and to embrace women as reliable stakeholders to share their experience of the MNH continuum.

## Conclusion

The inclusion of women’s feedback on their experiences of continuity for effective coordination of care in the MNH continuum revealed that their needs and preferences ought to be packaged in a way that the SBAs acknowledge them as potential reflections of continuity and coordination of care in the MNH continuum.
